# Revealing Structural Modifications of Lignin in Acidic γ-Valerolactone-H_2_O Pretreatment

**DOI:** 10.3390/polym12010116

**Published:** 2020-01-05

**Authors:** Suxiang Li, Chengke Zhao, Fengxia Yue, Fachuang Lu

**Affiliations:** 1 State Key Laboratory of Pulp and Paper Engineering, South China University of Technology, 381 Wushan Rd., Tianhe District, Guangzhou 510640, Chinamszck@foxmail.com (C.Z.); yuefx@scut.edu.cn (F.Y.); 2 Guangdong Engineering Research Center for Green Fine Chemicals, Guangzhou 510640, China

**Keywords:** γ-valerolactone, lignin, pretreatment, formaldehyde, 1,3-dioxane

## Abstract

γ-valerolactone (GVL)/H_2_O/acid solvent mixtures has been used in chemical pretreatment of lignocellulosic biomass, it was claimed that GVL lignins were structurally close to proto (native) lignins, or having low molecular weight with narrow polydispersity, however, the structural changes of GVL lignins have not been investigated. In this study, β-O-4 (β-aryl ether, **GG**), β-5 (phenylcoumaran), and β-β (resinol) lignin model compounds were treated by an acidic GVL-H_2_O solvent system, a promising pretreatment of lignocellulose for biomass utilization, to investigate the structural changes possibly related to the lignin involved. NMR characterization of the products isolated from the treated **GG** indicated that a phenyl dihydrobenzofuran, having typical C-H correlations at δ_C_/δ_H_ 50.74/4.50 and 93.49/4.60 ppm in its HSQC spectrum, was produced from **GG**. In the pretreatment, the released formaldehyde from **GG** reacted fast with **GG** to form a novel 1,3-dioxane intermediate whose characteristic HSQC signals were: δ_C_/δ_H_ 94.15–94.48/4.81–5.18 ppm and 80.82–83.34/4.50–4.94 ppm. The β-5 model, dihydrodehydrodiconiferyl alcohol, was converted into phenylcoumarone and stilbene having benzaldehyde that resulted from the allyl alcohol side chain. The β-β model, syringaresinol, was isomerized to form a mixture of syringaresinol, epi-, and dia-syringaresinol although being degraded slightly.

## 1. Introduction

Occurring in lignocellulose biomass, lignin is a natural amorphous polymer and a renewable resource to produce the chemicals, biomaterials, and biofuels [1,2]. The complex crosslink structure of lignin is one of the factors responsible to give its recalcitrance. Therefore, the bioconversion of cellulose to ethanol or other biofuels usually involves a pretreatment process to facilitate the follow up enzymatic saccharification [3].

In general, the pretreatment methods include biological, physical, chemical, and physicochemical methods. The organic solvent pretreatment methods have attracted attention because of the recycling utilization of solvents, low input, and the resultant high-quality lignins [4]. GVL is a compound that occurs naturally in fruits, and can be derived from carbohydrate biomass. Due to its high boiling (207 °C) and flashing point (96 °C), low melting point (−31 °C), and low vapor pressure (3.5 kPa under 80 °C), GVL is safe to be transported and stored [5]. These properties of GVL make it an excellent candidate used as an organic solvent for the pretreatment of biomass. In 2014 Luterbacher [6] reported the novel method of using GVL/H_2_O/H_2_SO_4_ (80−90 wt%/20−10 wt%/0.05 wt%) as a solvent system to pretreat corn stover, hardwood, or softwood. They found that the mixed solvent could almost dissolve the entire biomass constituent and GVL was recycled by the addition of NaCl or liquid CO_2_. Since then an increasing number of researchers have paid attention to using GVL for biomass pretreatments [7,8,9,10].

Understanding the structural changes of lignocellulose components, especially lignins, is imperative to identify a good pretreatment strategy or to optimize processing conditions and particularly to provide a basis for further utilization of lignins produced. However, it is not trivial to follow the structural changes of lignin in any chemical process because of the complicate structures of lignin and plant cell wall matrix. Thus, lignin model compounds representing substructures of lignin have often been used to investigate mechanisms of lignin reaction involved in many circumstances including the acid degradation.

During the period of 1967–1972, Lundquist et al. [11,12,13] investigated the reaction mechanisms of β-O-4 lignin model compounds and Björkman lignin from spruce under acidolysis conditions (90% 1,4-dioxane, and 0.2 mol/l HCl under reflux). Yasuda et al. [14,15,16,17] also proposed acidolysis mechanisms of β-O-4 lignin models. It was well documented that β-O-4 aryl ether models were degraded into guaiacol and Hibbert’s ketones via enol ethers intermediates formed from acid catalyzed dehydration of the 1,3-diol model; meanwhile the released guaiacol attacked the benzyl cation resulting in the condensation product (supporting information, Figure S1). Lundquist et al. [18,19,20,21] described the degradation mechanisms of phenylcoumaran type (β-5) models under acidolysis conditions. When phenolic or non-phenolic β-5 lignin models having propyl alcohol (R_1_ = CH_2_-CH_2_-CH_2_OH) or a ketone (R_1_ = CH_2_-CO-CH_2_OH) side chain, the acidolysis products were coumarone, stilbene, and formaldehyde, generated from the intermediate benzylium ion following the ring opening while the side chains were kept intact. Although the β-5 lignin model with allyl alcohol side chain (R_1_ = CH=CH-CH_2_OH) also gave coumarone and stilbene products under acidolysis condition, its allyl alcohol side chain was converted into ketone (R_1_ = CH_2_-CO-CH_2_OH) (Figure S2) in various yields depending on whether they were phenolic or not [20]. Resinol (β-β) lignin model was reported not degraded under acidolysis condition although it was partially isomerized into epi-pinoresinol [18].

In spite of [22] claiming that GVL lignins (obtained from pretreatment of lignocellulose in GVL solvent) were structurally close to proto (native) lignins, or having low molecular weight with narrow polydispersity, recent controversy results regarding GVL lignin was obtained (personal communication). Furthermore, there was no study with lignin models to understand the reactions of lignins during GVL pretreatment. This study here is to reveal the structural changes of lignins under GVL-H_2_O-H_2_SO_4_ pretreatment conditions with model compounds representing β-O-4, β-5, and β-β substructures of lignins as the reactants, by monitoring and characterizing the reaction intermediates or/and final products by GC-MS and 2D NMR. It was expected that the results of this work would provide new insights into the reaction mechanism of lignins involved in biomass pretreatment under such conditions helping to develop better process for utilizing the resultant lignin.

## 2. Materials and Methods 

### 2.1. Materials

Model compounds β-O-4 **GG**, β-5, and β-β were synthesized according to the reported methods [23,24,25,26]. GVL (98%) was purchased from Macklin (Shanghai, China), sulfuric acid was obtained from Guangzhou Chemical Reagent (Guangzhou, China). All chemicals were used as received without further purification. 

### 2.2. Acid Pretreatment of Lignin Models in GVL/ H_2_O (80/20) System

In order to monitor structural modification of lignin under acid pretreatment conditions, lignin model compounds, β-O-4 (**GG**), β-5, and β-β were subjected to acid treatment at 120 and 170 °C in a 10 mL glass pressured reaction tube with sulfuric acid (10 mM, ~0.1%) as a catalyst in GVL/H_2_O (80/20, wt/wt) solvent system. The starting material (model compounds) to liquor ratio was 1:15 g/mL. Each reaction mixture produced in various given times was cooled down (ice-water bath) to room temperature quickly, then added ethyl acetate and internal standard to extract the products and calculate the yields of products. The organic phase was washed with water (twice), then dried over saturated NaCl and anhydrous MgSO_4_. Filtered through a sintered glass funnel, the ethyl acetate finally was evaporated under reduced pressure at 43 °C. The reaction mixtures after evaporation were analyzed by a gas chromatograph coupled with mass spectrometer (GC-MS), or/and separated by silica-gel liquid chromatography using ethyl acetate/hexane as an eluent and TLC (Thin layer chromatography, silica gel 60 F_254_). The isolated products were characterized by ^1^H and ^13^C NMR, as well as 2D HSQC, HMBC, and COSY experiments.

### 2.3. GC-MS Measurements

Each 10 mg reaction mixtures with corresponding amounts of standard compound were dissolved into 800 μL ethyl acetate in a GC vial, then pyridine (100 μL) and N,O-bis trimethylsilyl trifluoroacetamide (BSTFA, 98%, 100 μL) were added, the mixtures were trimethylsilyl derivatized at 50 °C and kept for 40 min. At last, the mixtures were analyzed by a GC-MS-TQ-instrument (Shimadzu GCMS-TQ8040 triple quadrupole GC/MS/MS, Kyoto, Japan) equipped with a SH-Rxi-5Sil MS column (Shimadzu, 30 m × 0.25 mm × 0.25 μm).

### 2.4. NMR Measurements

NMR spectra were recorded in acetone-d_6_ or DMSO-d_6_ using a Bruker AVANCE Ⅲ HD 600 MHz spectrometer, the central acetone solvent peak δ_ppm_ (29.80, 2.04) and DMSO solvent peak δppm (39.50, 2.49) were used as an internal standard reference.

### 2.5. Synthesis of 1,3-Dioxane Compound 1 

The 1,3-dioxane compounds were synthesized according to the published method [27] with slight modifications: 100 mg **GG** was added to a 15 mL glass vial, followed by 3.6 mL of 1,4-dioxane, 168 μL of an HCl solution (37 wt%), and 400 μL of a formaldehyde (FA) solution (36.5 wt%). The mixture was stirred and kept at 80 °C for 30 min, and then neutralized with NaHCO_3_ (~168 mg in 20 mL water) and evaporated to dryness under reduced pressure. The resultant products were extracted with ethyl acetate and the organic solvent was washed with saturated aqueous NaCl, dried over anhydrous MgSO_4_, filtered through a sintered glass funnel, and finally dried under reduced pressure at 43 °C.

## 3. Results

### 3.1. Reactions of β-O-4 Lignin Model **GG** in GVL/ H_2_O (80/20) System

**Figure 1:**
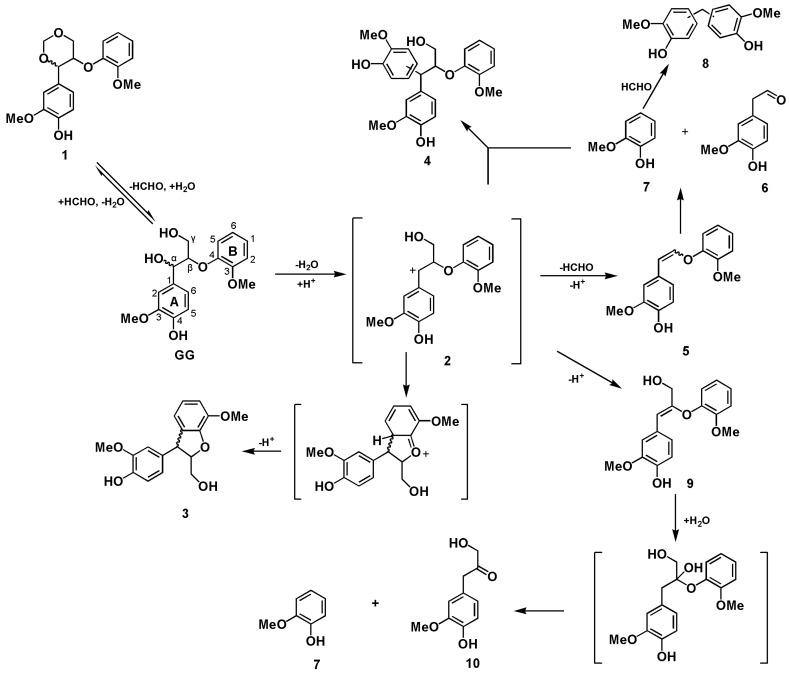
The proposed pathways of **GG** conversion into various products during the GVL-H_2_O-H_2_SO_4_ treatment.

When the lignin model **GG** was treated in an acidic GVL-H_2_O system at elevated temperature, the reaction media became brown and its color intensified with time. A dark substance insoluble in ethyl acetate was produced in 25 min suggesting that condensation (polymerization) reactions may happen during the process. The reactions of **GG** in the GVL-H_2_O-H_2_SO_4_ system were monitored by GC-MS. It was found that **GG** remained a lot in 1 h at 120 °C whereas it almost disappeared in 25 min at 170 °C. The phenyl dihydrobenzofuran **3** and 1,3-dioxane products **1** were isolated from a 15 min reaction at 170 °C and characterized by 2D NMR. The NMR spectra suggested their structural features similar to those of **3** and **1** when comparing to those reported in literatures [27,28]. The identity of 1,3-dioxane structures of compound **1** was finally confirmed by comparison with the synthesized (based on the reported method, see experimental) and authenticated reference. It was observed by the GC-MS that 1,3-dioxane compound **1** was formed in a 2 min reaction time while a trace amount of diphenylmethane **8** was detected in 10 min at 170 °C, suggesting that the formation of 1,3-dioxane structures **1** was easier than the formation of diphenylmethane **8** that was derived from guaiacol released by β-ether cleavage. In addition, the other compounds produced were the condensation product trimer **4**, enol ether **5**, homovanillin **6**, guaiacol **7,** and Hibbert’s Ketone **10** (Figure 1). 

**Figure 2:**
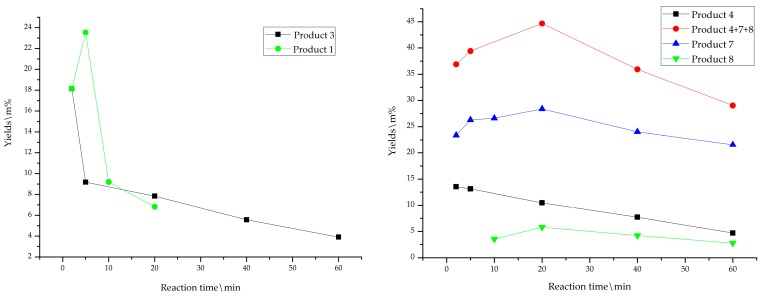
Changes in the content of products from β-O-4 model GG treated at 170 °C in GVL-H_2_O-H_2_SO_4._

To better understand the acid treatment process, reactions of **GG** in the GVL-H_2_O-H_2_SO_4_ system were monitored by GC-MS analysis of the major products formed in reaction times of 2, 5, 10, 20, 40, and 60 min and the product yields were shown in Figure 2. From the results shown in Figure 2, it was suggested that 1,3-dioxane structures **1** were produced in 2 min and their yields reached the maximum in 5 min following a quick decline, and then almost disappeared in 40 min. It should be mentioned that no such 1,3-dioxane products were ever reported from **GG** under conventional acidolysis conditions [11,12,13,14,15,16,17,18,29]. To better understand the fate of 1,3-dioxane intermediate compound **1**, the synthesized **1** was subjected to the acidic treatment in an aqueous GVL solvent. It was found that **GG** and its degradation products, i.e., enol ether structures **5**, phenyl-dihydrobenzofuran **3**, and condensation product **4** were detected in 5 min (Figure S14). However, the enol ether structures **5** and **GG** disappeared whereas 1,3-dioxane **1** still remained in 25 min at 170 °C. Within 25 min the yields of phenyl-dihydrobenzofuran **3** and condensation product **4** increased with time at 170 °C while **GG** was degraded gradually. Therefore, it can be speculated that the transformations between **GG** and 1,3-dioxane **1** was a reversible process; **GG** was more likely to be converted to other products whereas 1,3-dioxane **1** tends to go back to **GG** releasing a formaldehyde (Figure 1). 

The yields of phenyl-dihydrobenzofuran **3** reached the maximum in 2 min and declined quickly in the late stage according to GC-MS analysis, suggesting that **3** could be produced easily but was not stable at 170 °C (Figure 2). The mechanism for **GG** being converted to **3** (in 0.2 M H_2_SO_4_ at 150 °C in water and dioxane and ethylene glycol mixture) [30,31], was reported as shown in Figure 1. The stabilized benzylic carbocation intermediate **2** is formed by dehydration of the protonated α-hydroxyl group under the acidolysis conditions, and intramolecular Friedel-Crafts-type alkylation produces the intra-condensation product phenyl-dihydrobenzofuran **3** (Figure 1).

Being electron donating substitutes, both methoxyl and phenolic hydroxyl groups make the aromatic A-ring rich in the electron so that the benzylic carbonium **2** is highly stabilized in addition to the beneficial effect from the hydroxymethyl group (γ-carbon). Moreover, GVL, a polar aprotic solvent, is also helpful for the formation and stabilization of intermediate **2** [32]. On the other hand, the methoxyl and phenolic hydroxyl on the B-ring make the guaiacyl unit an excellent nucleophile and they both compete for directing nucleophilic sites on the ring. Overall, the C5 position of the B ring is favored in terms of geometry and electron density (**GG**). All of the above are possible reasons for the formation of phenyl-dihydrobenzofuran **3** in the acidic GVL-H_2_O system (Figure 1).

**Figure 3:**
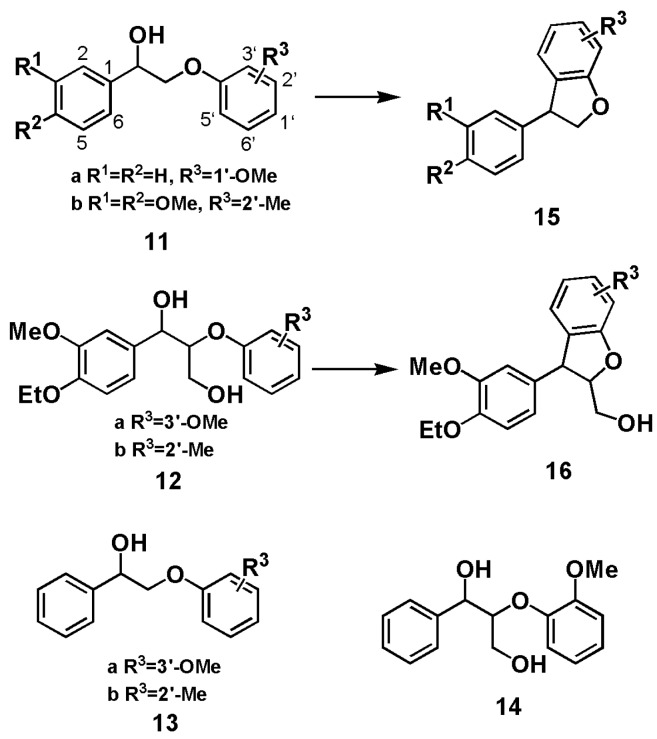
Lignin model compounds and the corresponding phenyl-dihydrobenzofuran formed in dioxane and ethylene glycol mixture at 140 °C with microwave radiation [28].

**Figure 4:**
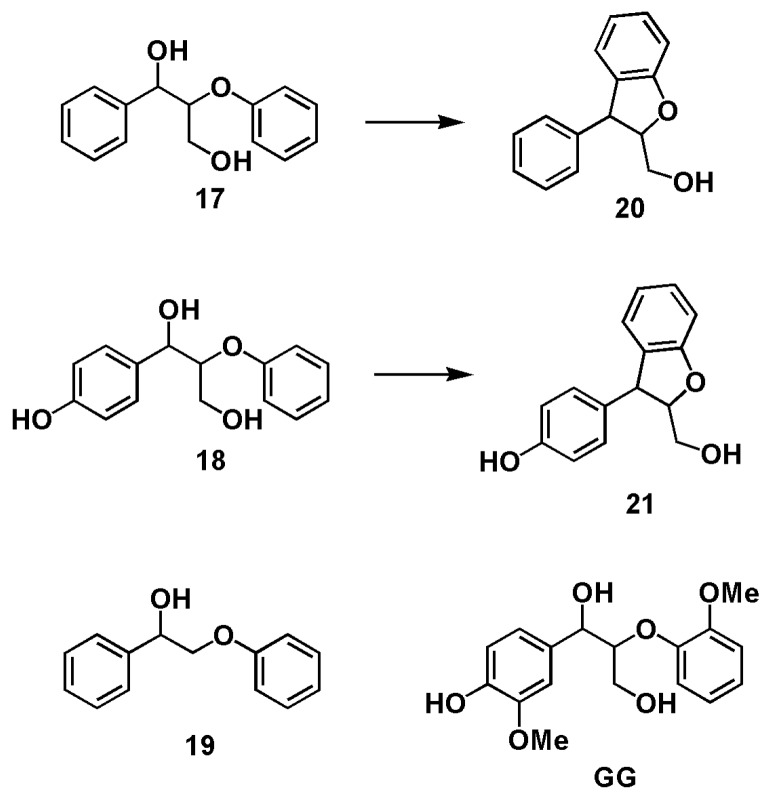
Lignin model compounds and the corresponding phenyl-dihydrobenzofuran formed in 0.2 M H_2_SO_4_ at 150 °C in water [31].

It is interesting to note in [28] that related model compounds **11** and **12**, in an acidic aqueous dioxane/ethylene glycol mixture at 140 °C heated by microwave radiation gave rise to the formation of phenyl-dihydrobenzofurans **15** and **16** whereas compounds **13** and **14** failed to form any phenyl-dihydrobenzofuran products (Figure 3). In another report [31], lignin models (1-phenyl-2-phenoxy-1,3-propanediol) **17** and (1-(4-hydroxyphenyl)-2-phenoxy-1,3-propane-diol) **18** were transformed to phenyl-dihydrobenzofurans **20** and **21,** respectively under acidolysis condition (aqueous 0.2 M H_2_SO_4_ at 150 °C) whereas model compounds **19** and **GG** could not produce the corresponding phenyl-dihydrobenzofuran products (Figure 4). However, as demonstrated here, from **GG** under acidic GVL/H_2_O condition the phenyl-dihydrobenzofuran **3** was produced readily. Comparing these reaction systems mentioned above draws the conclusion that reaction solvents and structures of model compounds dictate the formation of phenyl-dihydrobenzofuran products.

The aryl enol ether **9** is usually considered as an intermediate produced from acidolysis of both phenolic and non-phenolic β-O-4 lignin model compounds in ethanol-water with HCl or AlCl_3_ as catalysts, in 1-butyl-3-methylimidazolium chloride ([BMIM]Cl)/water with metal chlorides, and in 1-H-3-methylimidazolium chloride/water system. It was hydrolyzed to Hibbert’s Ketones including ketone **10** and guaiacol immediately, although usually it was not detected due to its instability under such acid conditions [33,34,35]. In addition to the formation of Hibbert’s Ketones and guaiacol, it was also reported that the enol ether **5** and aryl acetaldehydes are formed under various acidolysis conditions (0.2 M HBr, 0.2 M HCl, and 0.1 M H_2_SO_4_ in 82% aqueous 1,4-dioxane at 85 °C, and 0.2 M H_2_SO_4_ at 150 °C in water) [36,37]. 

The formation of homovanillin **6** and guaiacol **7** were likely to proceed through a mechanism similar to the one reported in the literatures [27,30,31,32,33], i.e., the α-hydroxyl group of **GG** model compound is protonated, then followed by dehydration to produce a benzylic carbonium **2**, then a formaldehyde was released from the β-position forming the enol ether **5** that was further degraded into homovanillin **6** and guaiacol **7** (Figure 1). Imai [37] found that the releasing of the formaldehyde from the β-carbon was the dominate reaction with sulfuric acid as a catalysis in acidolysis conditions. The current study also observed similar results, i.e., trace amount of Hibbert’s Ketone **10** was detected only in the early stage (Figure 2). 

As illustrated in Figure 1, guaiacol released from **GG** degradation could act as a reactant, undergoing subsequent reactions [38,39]. In one way, the released guaiacol attacked the carbonium **2** producing a trimer **4** [37] whose yield reached maximum in 5 min and then decreased slowly implying that a further transformation is possible. In another way, two molecules of guaiacol that reacted with one molecule formaldehyde produced the diphenyl methane **8** (Figure 1). According to the obtained results, the guaiacol released from **GG** existed in three kinds: trimer **4,** guaiacol **7,** and diphenyl methane **8**, the amount of the three products reached maximum in 20 min indicating that the **GG** compound was greatly modified or degraded (Figure 2). 

In summary, the formation of 1,3-dioxane structures **1** and phenyl-dihydrobenzofuran **3** from **GG** under the acidic GVL-H_2_O system suggests that the actions of GVL pretreatment on lignin model **GG** was different from those induced by the traditional acidolysis.

### 3.2. Reactions of β-5 Lignin Models in GVL/ H_2_O (80/20) System

**Figure 5:**
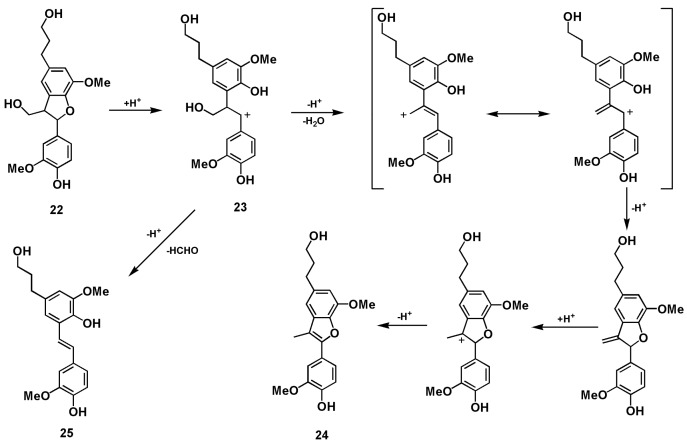
Proposed pathways of β-5 structure 22 conversions into various products during GVL-H_2_O-H_2_SO_4_ treatment.

**Figure 6:**
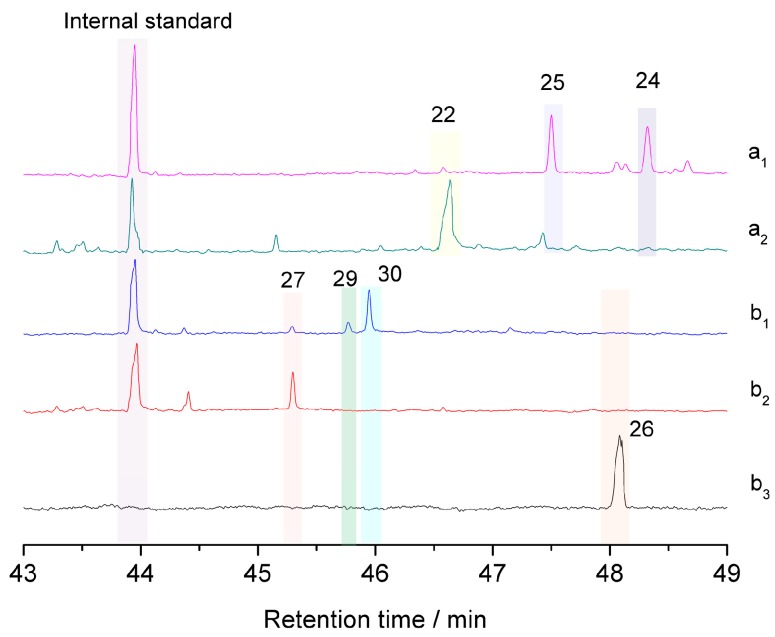
Total ion chromatography of the products after GVL-H_2_O-H_2_SO_4_ treatment at 170 °C obtained from β-5 models. a_1_: Products obtained from **22** in 60 min; a_2_: Products obtained from **22** in 2 min; b_1_: Products obtained from **26** in 60 min; b_2_: Products obtained from **26** in 2 min; b_3_: Lignin model compound **26**.

**Figure 7:**
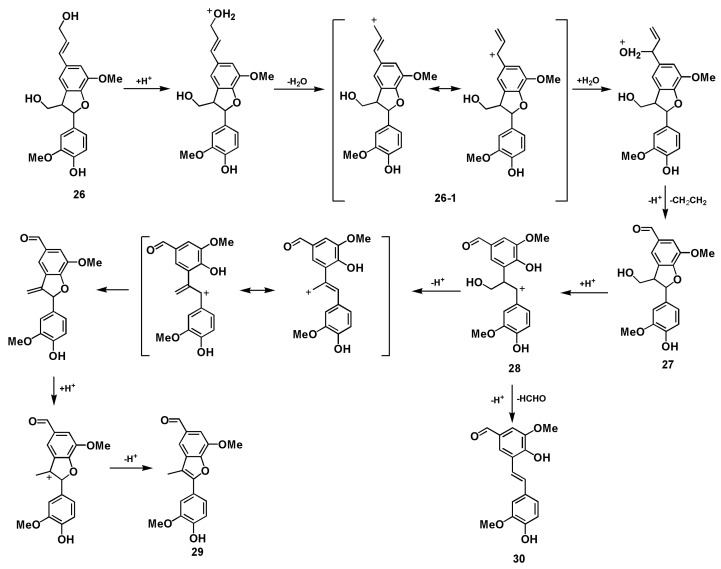
Proposed pathways of β-5 structure **26** conversions into various products during GVL-H_2_O-H_2_SO_4_ treatment.

The β-5 (phenylcoumaran) types of structures are the second most abundant component of lignin. The reactions of β-5 lignin model compounds **22** and **26** have been investigated under acidolysis conditions [18,19,20,21]. In the acidic GVL-H_2_O system used here, compound **22** was transformed following the pathway described in Figure 5. The side chain remained unchanged and the stilbene **25** was produced through intermediate benzylic cation **23**, from which dehydration and rearrangement occurred. Meanwhile, the coumarone **24** was formed as a sequence of ring-opening, allylic rearrangement following the elimination of β-proton and water, and re-cyclization, finally the exocyclic styrenoid double bond migrated into the more suitable stilbenoid position under the acidic condition [20] (Figure 5, Figures S15–S18). However, when dihydrodehydrodiconiferyl alcohol **26** was treated under the same condition, coumaran **27** was mainly produced quickly in 2 min when no starting material **26** was left suggesting that the allyl alcohol moiety was degraded while the dihydrobenzofuran ring remained. After 60 min treatment, phenylcoumarone **29** and stilbene **30** were produced although small amounts of **27** were still detected (Figure 6, Figures S19–S23). As mentioned above, GVL is helpful for the formation and stabilization of benzylic carbonium intermediate **2**, it is believable that GVL also contributes to the formation and stabilization of conjugated system **26-1**, thereafter **27** could be formed in acidic aqueous GVL solvent. As described in Figure 7, the intermediate benzylic carbonium **28** was generated through opening of the dihydrobenzofuran ring of **27**, and the coumarone **29** was produced in a way similar to that of **24** in one direction whereas formaldehyde was released from **28** producing stilbene **30** in another direction. 

Overall, the modification of β-5 structures without allyl alcohol of lignins in acidic GVL-H_2_O system was similar to that found in the traditional acidolysis conditions. However, the allyl alcohol group of β-5 structures was transformed into a benzaldehyde group in the acidic GVL-H_2_O system (Figure 6) whereas it was converted to Hibbert’s ketone in traditional acidolysis.

### 3.3. Reactions of β-β Lignin Model in GVL/ H_2_O (80/20) System

**Figure 8:**
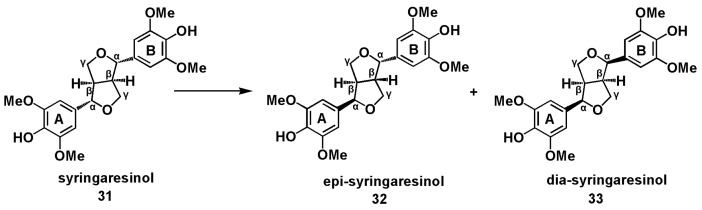
Isomers obtained from the isomerization of syringaresinol.

Unlike β-O-4 and β-5 model compounds, the β-β model pinoresinol is relatively stable under acidolysis condition (0.2 M HCl in 9:1 dioxane-water solution) besides isomerization to epi-pinoresinol (50% yields) after refluxing for 4 h [18]. In this study, lignin β-β model compound syringaresinol was treated at 170 °C in the acidic GVL-H_2_O system. GC-MS analysis of the crude products indicated that the major compounds detected were isomers of syringaresinol (epi-syringaresinol, and dia-syringaresinol), which were confirmed by comparing their 2D NMR data with those reported in [40,41] (Figure 8 and Figure S24). Only a small amount of other unknown products were observed (Figure S24). The ratio of syringaresinol, epi-syringaresinol, and dia-syringaresinol was about 2.4:2:0.3 as estimated by ^1^H NMR after 60 min reaction under the acidic GVL-H_2_O system.

The products from syringaresinol treated at 170 °C in acidic GVL-H_2_O system were measured by GC-MS using an internal standard (Figure S3) and the total yields of the isomerized products were listed in Table 1. It can be seen that the yield of isomers was decreased with reaction time. Therefore, it is reasonable to speculate that β-β model compound was also degraded or condensed, although in a relatively slow speed, in acidic GVL-H_2_O system.

Based on the results from this model study, it is reasonable to expect that structures of lignins were modified greatly in such an acidic GVL-H_2_O system although the heterogeneity in lignin and cell wall matrix may lead to some variations from results observed here. That the reported GVL lignins were structurally close to proto (native) lignins probably was the result caused by incomplete assignment of the spectra of the sample due to lack of enough model studies. To solve such a controversy, it would be worthy to re-evaluate or analyze GVL-lignins from lignocelluloses based on the results of this model studies. 

## 4. Conclusions

The transformations of three kinds of lignin model compounds, phenolic β-O-4 model **GG**, β-5 (phenylcoumaran), and β-β (syringaresinol), in the GVL/H_2_SO_4_/H_2_O system at elevated temperatures (120–170 °C) have been examined. It was shown that β-O-4 and β-5 were completely converted producing a variety of products under such conditions (10 mM H_2_SO_4_ in acidic GVL-H_2_O system at 120–170 °C) while the β-β structure was relatively stable besides isomerization under the same condition. The aryl β-ether model **GG** was converted very quickly producing aryl 1,3-dioxane intermediate product, degradation products (homovanillin, guaiacol and Hibbert’s Ketone), condensation products (a trimer, diphenylmethane, and phenyl dihydrobenzofuran), as well as polymerized product (dark substance insoluble in ethyl acetate). The treatment of **GG** model in acidic GVL-H_2_O system used produced products (phenyl dihydrobenzofuran and aryl-1,3-dioxane structures) not found under aqueous acidolysis conditions. The β-5 lignin model (dihydrodehydrodiconiferyl alcohol) was converted into phenylcoumarone and stilbene products having benzaldehyde groups that resulted from the allyl alcohol, however, the resinol (β-β) model, syringaresinol, was partially isomerized to its epi- and dia-isomers. Quantitative analysis suggested that the resinol model was slowly degraded or condensed. 

## Figures and Tables

**Table 1:** Yields of products from of syringaresinol treated at 170°C in GVL-H_2_O-H_2_SO_4._

Reaction Time/min	20	40	60
Product (total isomers) yields (%)	95.8	85.5	61.2

## Supplementary Materials

The following are available online at https://www.mdpi.com/2073-4360/12/1/116/s1.
