# Supplementary material for: Revealing Structural Modifications of Lignin in Acidic γ-Valerolactone-H2O Pretreatment
Source: Polymers (Basel). 2020 Jan 5;12(1):116. doi: 10.3390/polym12010116 (PMC7023100; doi:10.3390/polym12010116)
Supplement: Supplementary file 1 [file polymers-12-00116-s001.pdf]

Supporting materials:

Revealing structural modifications of lignin in acidic γ-valerolactone-H_2O pretreatment

Suxiang Li[1], Chengke Zhao[1], Fengxia Yue[1], Fachuang Lu[1,2,*]

[1] State Key Laboratory of Pulp and Paper Engineering, South China University of Technology, 381 Wushan Rd., Tianhe District, Guangzhou, 510640, China. Email: fefclv@scut.edu.cn

[2]Guangdong Engineering Research Center for Green Fine Chemicals, Guangzhou, 510640, China.

[*]Corresponding author: Fachuang Lu, E-mail: fefclv@scut.edu.cn, Tel: +86-020-8711-3953

1. The reported mechanism of β-O-4 and β-5 substructures during acid treatment 3

2. Determination of Products from **GG** under acidic GVL solvent system.............................. 3

3. NMR data of products obtained from β-O-4 5

3.1 Synthesis and NMR Spectra of 1,3-dioxane structures (**1a, 1b**)................................. 5
3.2 NMR Spectra of phenyl dihydrobenzofuran **3** 7
3.3 NMR Spectra of condensation product trimer **4** 8
3.4 Synthesis and NMR Spectra of enol ether compounds (**5a, 5b**)................................. 9
3.5 NMR Spectra of Hibbert's Ketone **10** 10
3.6 TIC (total ion chromatogram) of the products obtained from β-O-4........................ 10
3.7 TIC (total ion chromatogram) of the products obtained from 1,3-dioxane compounds 11

4. Synthesis and NMR Spectra of products obtained from β-5...11

4.1 phenylcoumarone **24**...11
4.2 stilbene **25**.. 12
4.3 coumaran **27**... 13
4.4 phenylcoumarone **29**.. 14
4.5 stilbene **30**.. 15

5. Products obtained from syringaresinol (β-β).. 16

5.1 syringaresinol... 16
5.2 epi-syringaresinol... 16
5.3 dia-syringaresinol... 16
5.4 GC-MS of the products obtained from β-β.. 18

1. The reported mechanism of β-O-4 and β-5 substructures during acid treatment

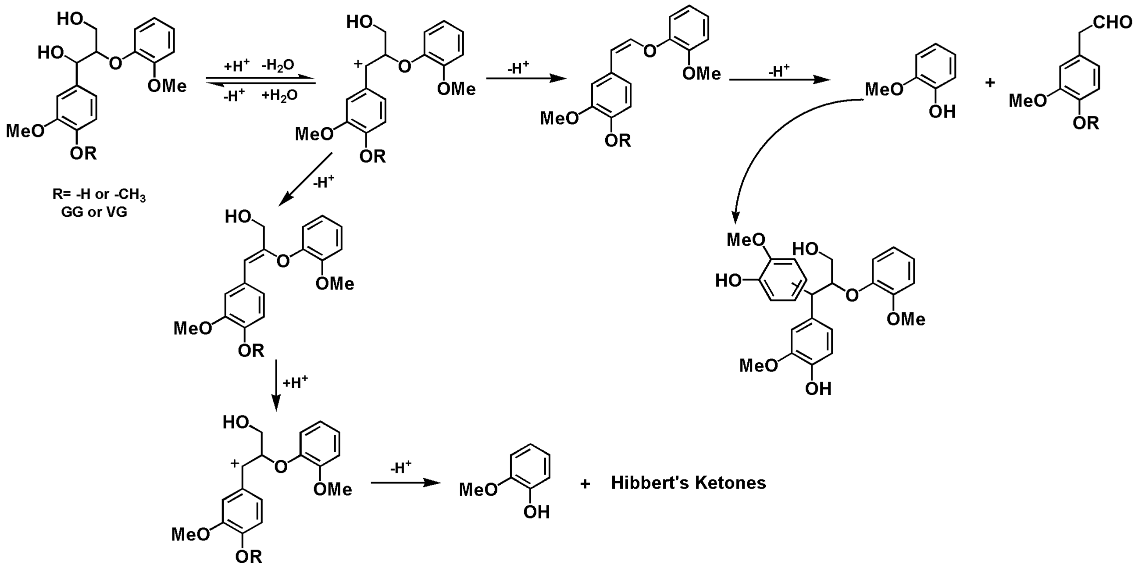

Fig.S1 The reported mechanism of β-O-4 substructure during acid treatment[1-10]

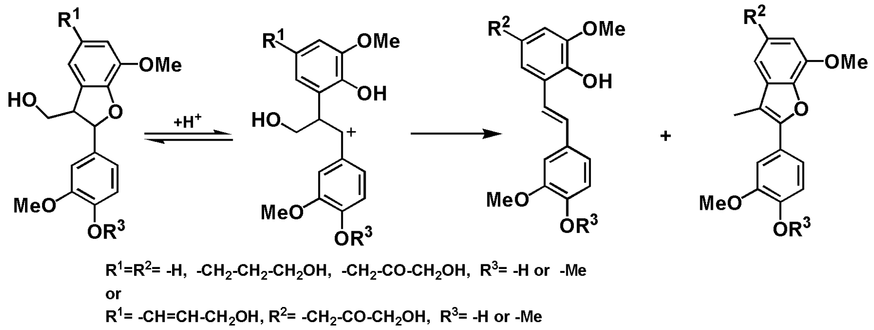

Fig.S2 The reported mechanism of β-5 substructure during acid treatment[11-15]

2. Determination of Products from GG under acidic GVL solvent system

The yields of products from GG were determined by GC-MS with internal standard (VG-dimer). Therefore, yields of each product were calculated by the following Eq.

$$Y = \frac{m_{AP} \times M_{IS}}{m_{IS} \times M_L} \times 100\%$$

Y: Yield of product (%);

m_{AP}: Mass weight of product for GC-MS analysis;

m_{IS} : Mass weight of internal standard for GC-MS analysis;

$M_{IS.}$: Mass weight of internal standard added;

M_L: Mass of lignin model.

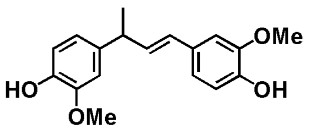

Fig.S3 Internal standard compound

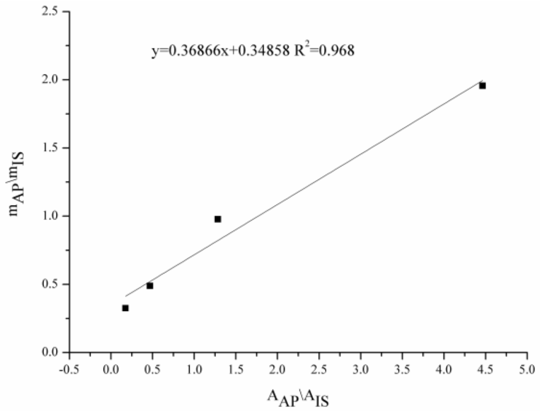

a)

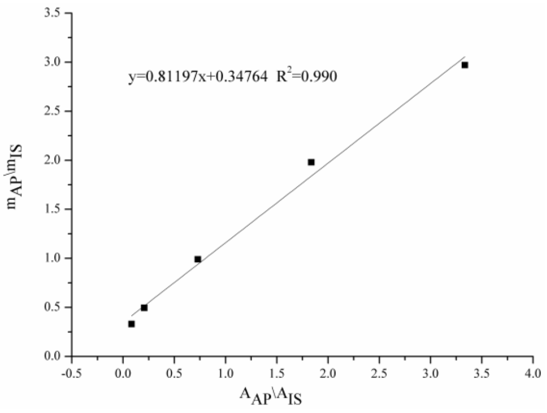

b)

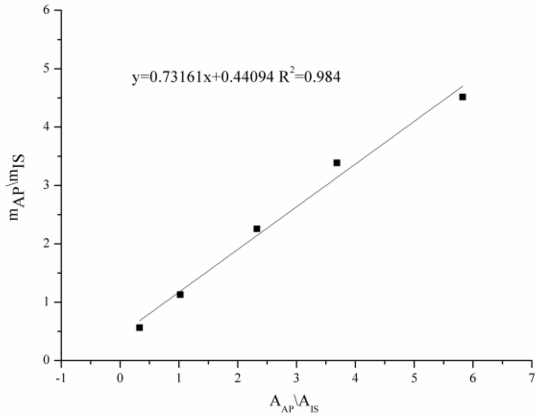

c)

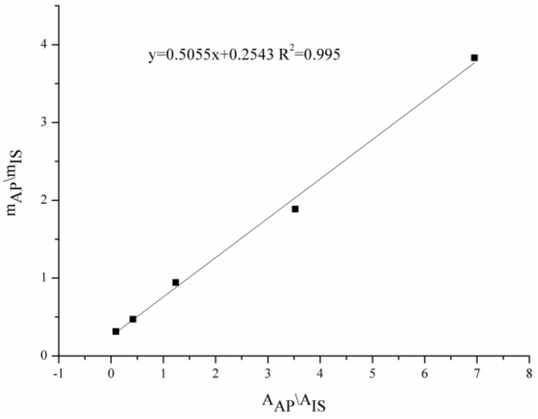

d)

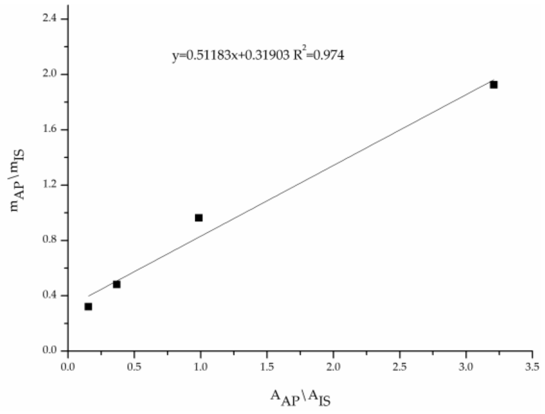

e)

Fig.S4 The standard curve of different product a)product 3, b)product 1, c)product 7, d)product 4, and e)product 8. A_{AP}: peak area of product; A_{IS}: peak area of internal standard.

3. NMR data of products obtained from β-O-4

3.1 Synthesis and NMR Spectra of 1,3-dioxane structures (1a, 1b)

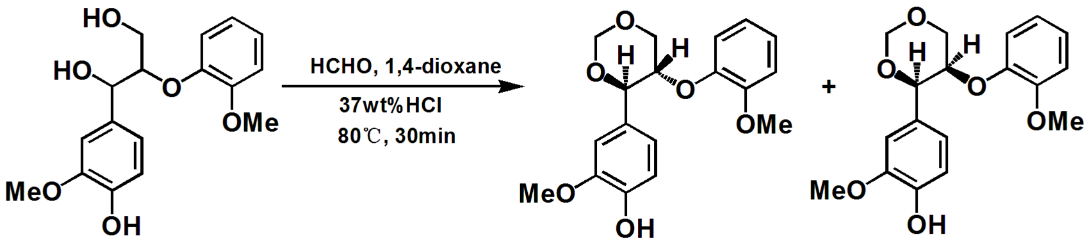

a b

1

Fig.S5 Synthesis route of 1,3-dioxane structures (GG)[16]

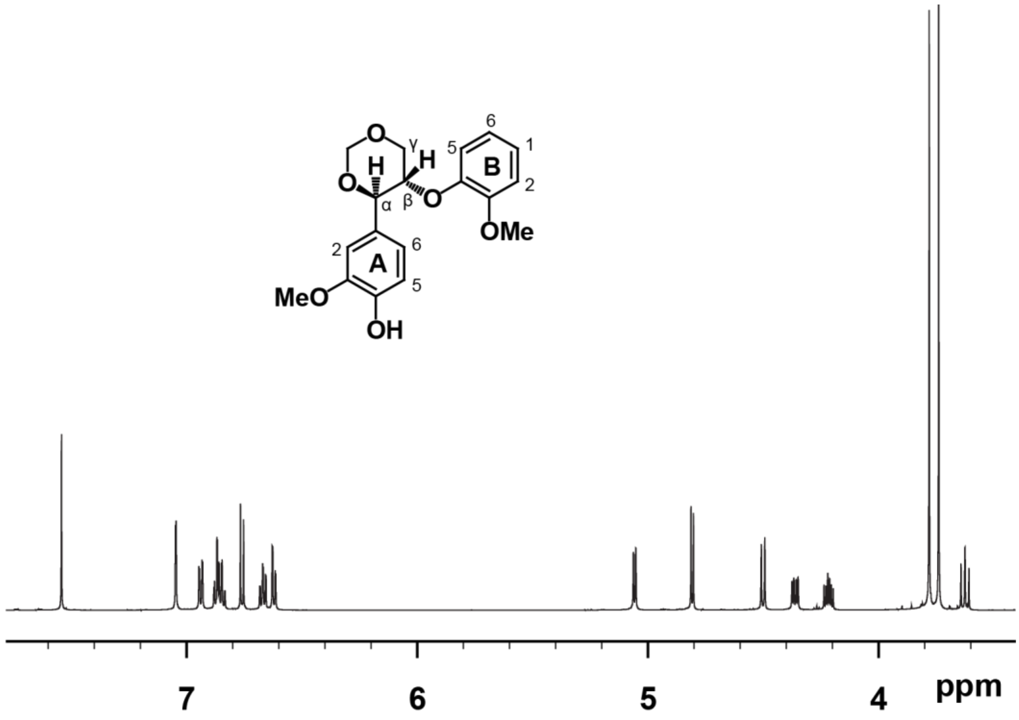

Fig.S6 ^{1}H NMR of trans-isomer of 1,3-dioxane structure, *trans*

1a, *trans*-isomer: ^{1}H-NMR (acetone-d6, 600MHz): δ 7.54 (s, 1H; ArOH), 7.05 (d, J=1.9 Hz, 1H; A2), 6.94 (dd, J=1.9, 8.1 Hz, 1H; A6), 6.87 (dd, J=1.9, 8.1Hz, 1H; B2), 6.85 (ddd, J=1.5, 6.9, 8.1Hz, 1H; B1), 6.76 (d, J=8.1 Hz, 1H; A5), 6.67 (m, 1H; B6), 6.62 (dd, J=1.5, 8.1Hz, 1H; B5), 5.06 (d, J=6.2 Hz, 1H; acetal-H), 4.81 (d, J=6.2 Hz, 1H; acetal-H), 4.50 (d, J=9.1 Hz, 1H; α-H), 4.36 (dd, J=5.0, 10.4 Hz, 1H; γ-H), 4.22 (ddd, J=5.0, 9.1, 10.4 Hz, 1H; β-H), 3.78 (s, 3H; OCH_3), 3.74 (s, 3H; OCH_3), 3.62 (t, J=10.4 Hz, 1H; γ-H).

^{13}C NMR (acetone-d6, 600MHz): δ151.69 (C-B3), 148.10 (C-A3), 147.97 (C-B4), 147.44 (C-A4), 131.20 (C-A1), 123.58 (C-B1), 121.57 (C-A6), 121.46 (C-B6), 118.80 (C-B5), 115.22 (C-A5), 113.61 (C-B2), 112.14 (C-A2), 94.15 (C-acetal), 83.34 (C-α), 75.66 (C-β), 70.07 (C-γ), 56.31 (C-OCH_3), 56.12 (C-OCH_3).

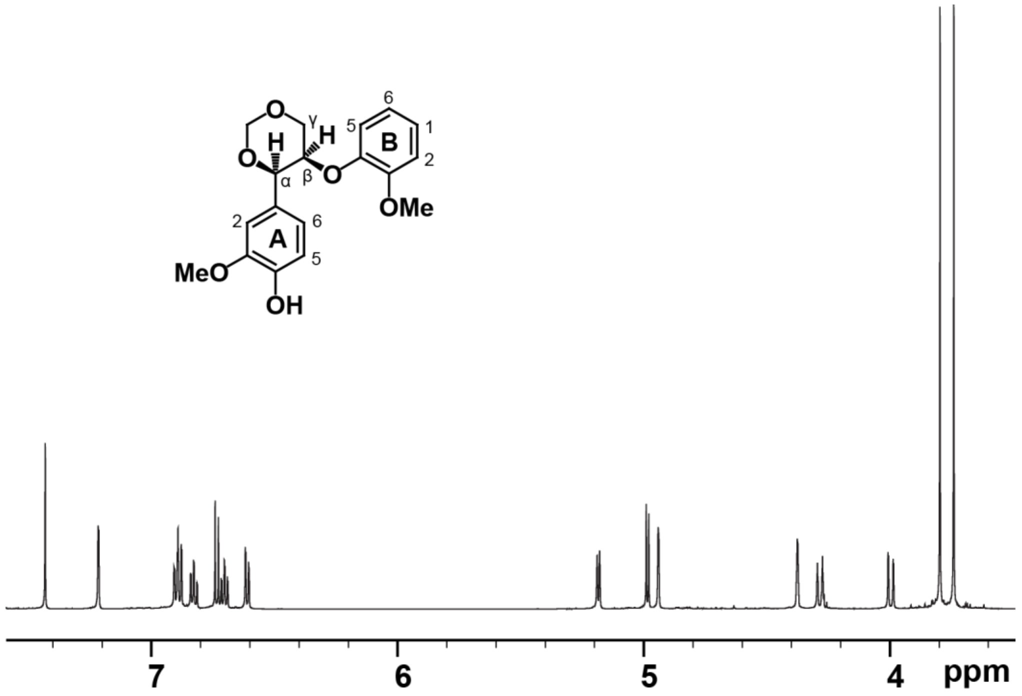

Fig.S7 ^{1}H NMR of cis-isomer of 1,3-dioxane structure, *cis*

1b, *cis*-isomer: ^{1}H-NMR (acetone-d6, 600MHz): δ 7.43 (s, 1H; ArOH-H), 7.21 (d, J=1.8 Hz, 1H; A2), 6.90 (dd, J=1.6, 8.2 Hz, 1H; A6), 6.88 (dd, J=1.5, 8.1Hz, 1H; B2), 6.83 (dt, J=1.5, 7.5, 8.1Hz, 1H; B1), 6.73 (d, J=8.2 Hz, 1H; A5), 6.70 (dt, J=1.6, 7.5, 8.0Hz, 1H; B6), 6.61 (dd, J=1.5, 8.0Hz, 1H; B5), 5.18 (d, J=6.3 Hz, 1H; acetal-H), 4.98 (d, J=6.3 Hz, 1H; acetal-H), 4.94 (d, J=1.5 Hz, 1H; α-H), 4.37 (d, J=1.5 Hz, 1H; β-H), 4.28 (dt, 1H; γ-H), 4.00 (dd, J=1.5, 12.4 Hz, 1H; γ-H), 3.80 (s, 3H; OCH_3), 3.74 (s, 1H; OCH_3).

^{13}C NMR (acetone-d6, 600MHz): δ151.85 (C-B3), 148.45 (C-B4), 147.85 (C-A3), 146.88 (C-A4), 131.24 (C-A1), 122.77 (C-B1), 121.63 (C-B6), 120.44 (C-A6), 118.15 (C-B5), 114.88 (C-A5), 114.25 (C-B2), 112.18 (C-A2), 94.48 (C-acetal), 80.82 (C-α), 75.02 (C-β), 68.71 (C-γ), 56.41 (C-OCH_3), 56.24 (C-OCH_3).

3.2 NMR Spectra of phenyl dihydrobenzofuran 3

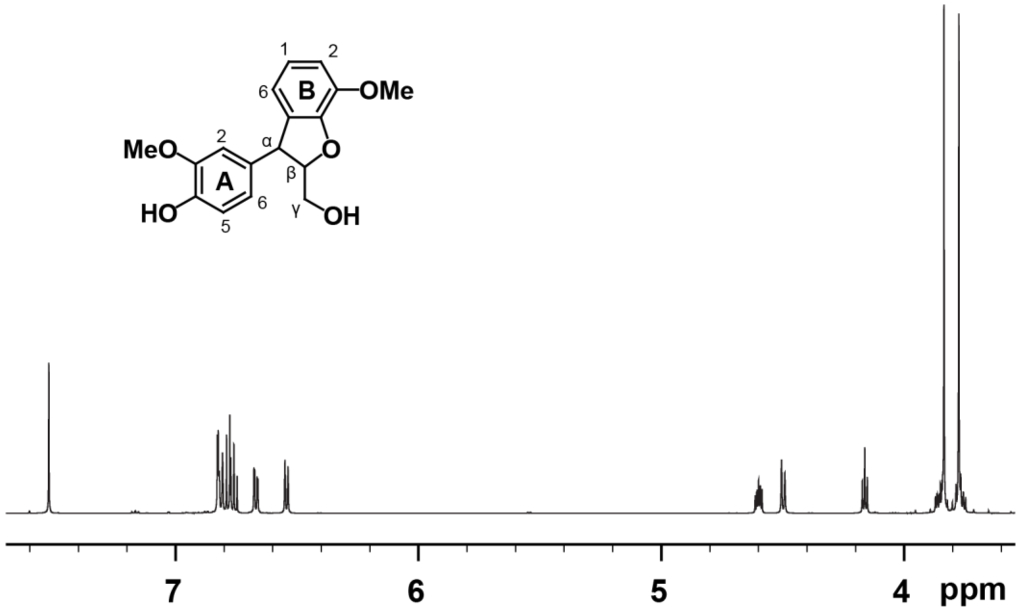

Fig.S8 ^{1}H NMR of phenyl dihydrobenzofuran **3** [17]

3, ^{1}H-NMR (acetone-d6, 600MHz): δ 7.52 (s, 1H; ArOH-H), 6.83 (d, J=2.0 Hz, 1H; A2), 6.81 (d, J=8.0 Hz, 1H; B2), 6.78 (d, J=8.1 Hz, 1H; A5), 6.76 (dd, J=8.0,7.6 Hz, 1H; B1), 6.67 (dd, J=8.1, 2.0 Hz, 1H; A6), 6.54 (dt, J=7.6Hz, 1H; B6), 4.61-4.58 (m, 1H; β-H), 4.50 (d, J=8.1 Hz, 1H; α-H), 4.16 (t, 1H; γOH-H), 3.86 (m, 1H; γ-H), 3.84 (s, 3H; OCH_3), 3.77 (s, 3H; OCH_3), 3.77 (m, 1H; γ-H).

^{13}C NMR (acetone-d6, 600MHz): δ 149.26 (C-B4), 148.49 (C-A3), 146.49 (C-A4), 145.44 (C-B3), 134.88 (C-A1), 133.03 (C-B5), 121.83 (C-B1), 121.48 (C-A6), 118.07(C-B6), 115.93 (C-A5), 112.73 (C-B2), 112.34 (C-A2), 93.49 (C-β), 63.40 (C-γ), 56.20 (C-OCH_3), 56.14 (C-OCH_3), 50.74 (C-α).

3.3 NMR Spectra of condensation product trimer 4

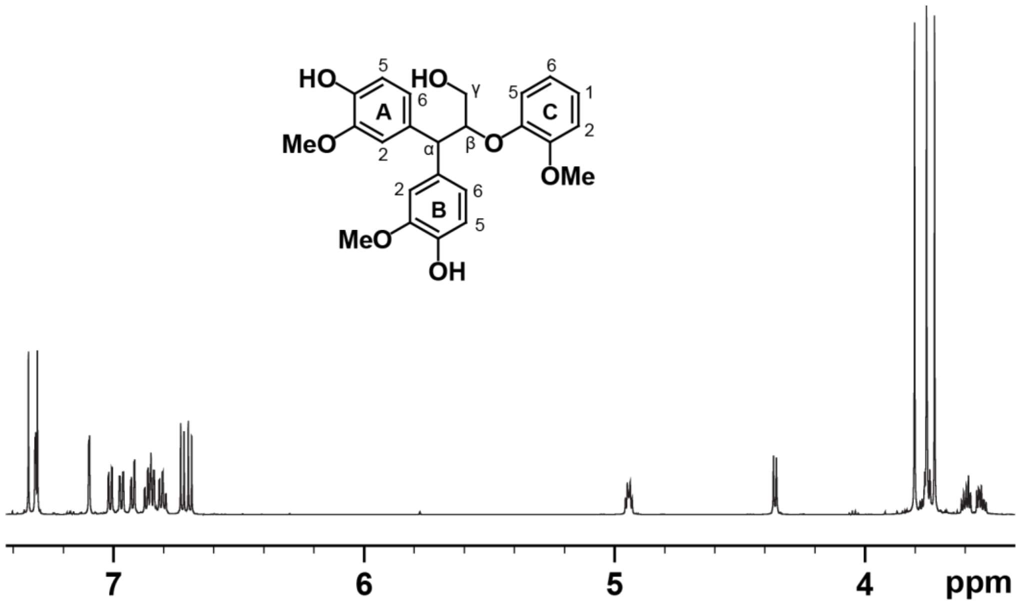

Fig.S9 ^{1}H NMR of trimer **4**

4, ^{1}H-NMR (acetone-d6, 600MHz): δ7.34 (s, 1H; ArOH-H), 7.31 (d, J=2.0 Hz, 1H; A2), 7.30 (s, 1H; ArOH-H), 7.10 (d, J=2.0 Hz, 1H; B2), 7.01 (dd, J=1.5, 8.0 Hz, 1H; C5), 6.97 (dd, J=2.0, 8.0 Hz, 1H; A6), 6.92 (dd, J=1.5,8.0Hz, 1H; C2), 6.86 (dt, 1H; C1), 6.84 (dd, J=2.0,6.5 Hz, 1H; B6), 6.80 (dt, J=1.6,7.8Hz, 1H; C6), 6.72 (d, J=8.0 Hz, 1H; A5), 6.69 (d, J=8.0 Hz, 1H; B5), 4.97-4.93 (m, 1H; β-H), 4.36 (d,J=6.9Hz, 1H; α-H), 3.80 (s, 3H; OCH_3), 3.75 (s, 3H; OCH_3), 3.72 (s, 3H; OCH_3), 3.62-3.57(m, 1H; γ-H), 3.77-3.51 (m, 1H; γ-H).

^{13}C NMR (Acetone-d6, 600MHz): δ 151.43 (C-C3), 149.18 (C-C4), 148.02 (C-B3), 147.86 (C-A3), 145.80 (C-B4), 145.76 (C-A4), 135.54 (C-B1), 134.44 (C-A1), 122.85 (C-A6), 122.40 (C-C1), 121.89 (C-B6), 121.75 (C-C6), 117.30 (C-C5), 115.47 (C-B5), 115.16 (C-A5), 113.81 (C-A2), 113.39 (C-C2), 112.94 (C-B2), 84.13 (C-β), 62.12 (C-γ), 56.26 (C-OCH_3), 56.17 (C-OCH_3), 56.07 (C-OCH_3), 52.56 (C-α).

3.4 Synthesis and NMR Spectra of enol ether compounds (5a, 5b)

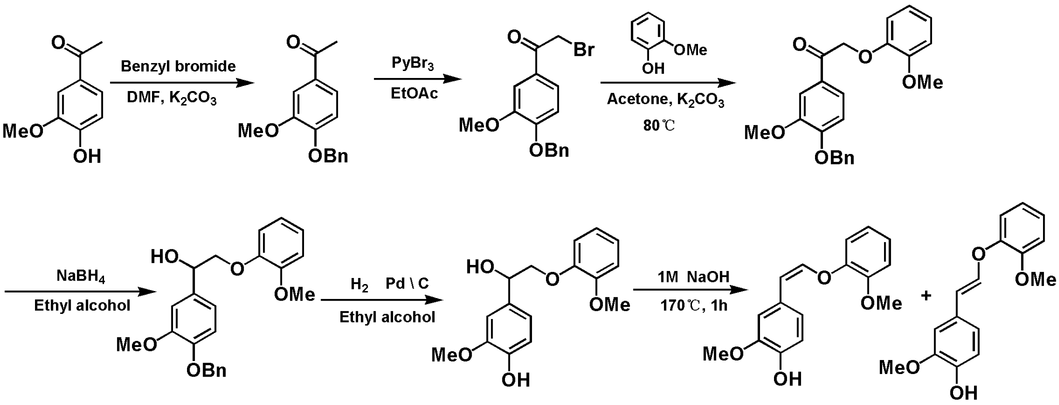

a b

5

Fig.S10 Synthesis route of (E/Z)-4-hydroxy-3-methoxy-β-(2-methoxyphenoxy)styrene (enol ether compounds **5a-b**)

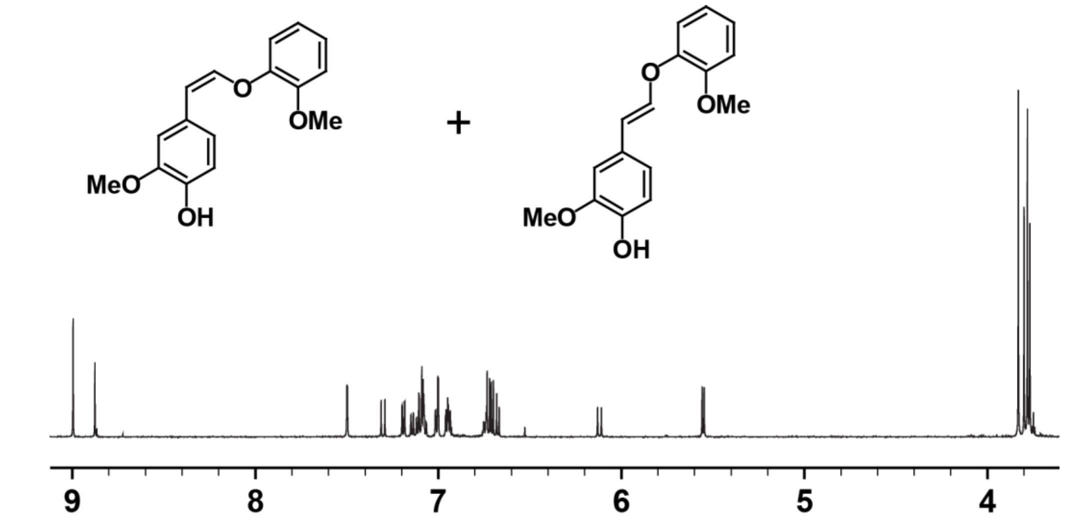

Fig.S11 ^{1}H NMR of enol ether compounds **5a-b**

5a, Z-isomer: ^{1}H NMR (DMSO-d6, 600MHz): δ 8.99 (s, 1H; ArOH-H), 7.50 (d, 1H; Ar-H), 7.18 (dd, 1H; Ar-H), 7.11 (dd, 1H; Ar-H), 7.07 (td, 1H; Ar-H), 7.01 (dd, 1H; Ar-H), 6.94 (td, 1H; Ar-H), 6.72 (d, 1H; Ar-H), 6.69 (d, 1H; β-H), 5.55 (d, 1H; α-H), 3.83 (s, 3H; OCH_3), 3.78 (s, 3H; OCH_3).

5b, E-isomer: ^{1}H NMR (DMSO-d6, 600MHz): δ 8.87 (1H, s, H-ArOH), 7.30 (1H, d, H-βH), 7.14 (1H, d, H-ArH), 7.09 (1H, dd, H-ArH), 7.08 (1H, td, H-ArH), 7.01 (1H, dd, H-ArH), 6.94 (1H, td, H-ArH), 6.74 (1H, dd, H-ArH), 6.67 (1H, d, H-ArH), 6.12 (1H, d, H-αH), 3.80 (3H, s, H-OCH_3), 3.77 (3H, s, H-OCH_3).

3.5 NMR Spectra of Hibbert's Ketone 10

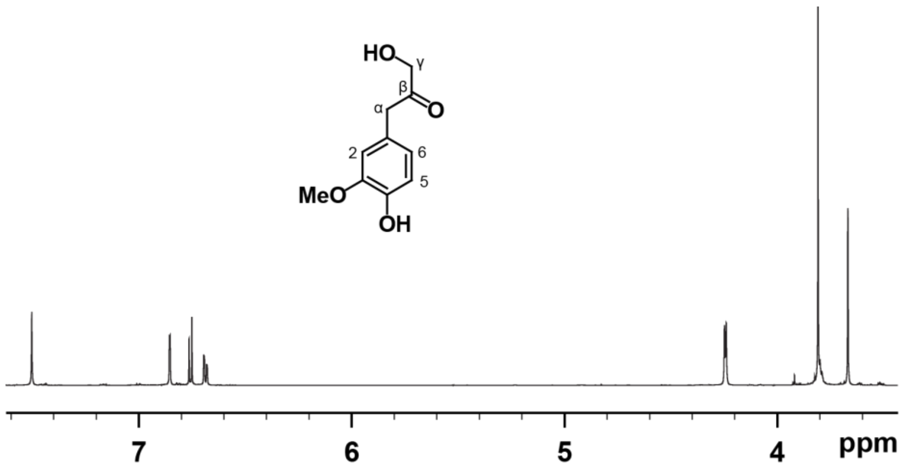

Fig.S12 ^{1}H NMR of Hibbert's Ketone **10**

10, ^{1}H-NMR (acetone-d6, 600MHz): δ 7.54 (s, 1H; ArOH-H), 6.85 (d, J=2.0 Hz, 1H; 2-H), 6.75 (d, J=8.0Hz, 1H; 5-H), 6.68 (dd, J=2.0, 8.0Hz, 1H; 6-H), 4.24 (s, 1H; γ-H), 3.87-3.81 (m, 1H; OH-H), 3.81 (s, 1H; OCH_3), 3.67 (s, 1H; α-H).

^{13}C NMR (acetone-d6, 600MHz): δ 208.68 (C-β), 148.29 (C-3), 146.45 (C-4), 126.24 (C-1), 122.92 (C-6), 115.77 (C-5), 113.86 (C-2), 68.08 (C-γ), 45.35 (C-α), 56.18 (C-OCH_3).

3.6 TIC (total ion chromatogram) of the products obtained from β-O-4

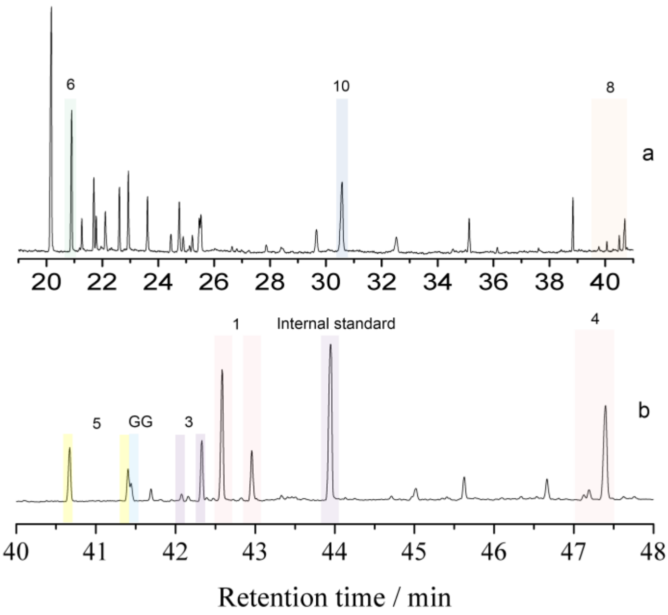

Fig.S13 GC-MS of the products obtained from β-O-4 at 170°C after GVL-H_2O-H_2SO_4 treatment a) to show products **6**, **8** and **10** were produced in 10 min reaction; b) to show products **1**, **3**, **4** and **5** were produced in 5 min reaction.

3.7 TIC (total ion chromatogram) of the products obtained from 1,3-dioxane compounds

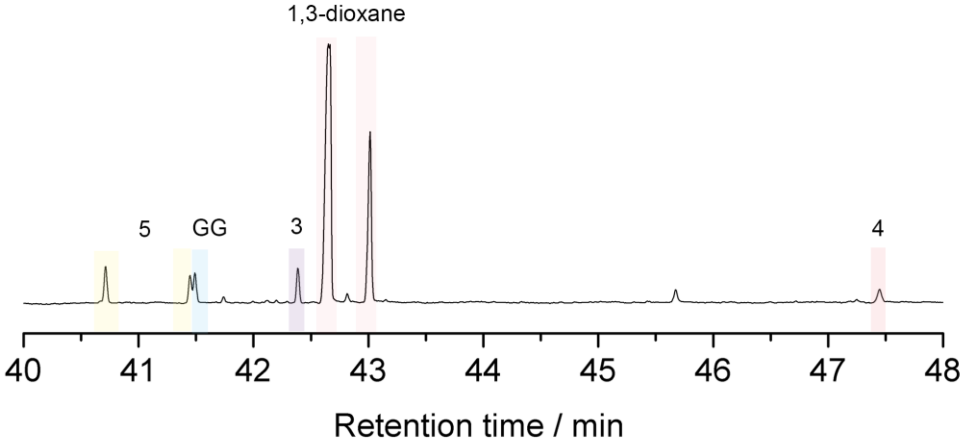

Fig.S14 GC-MS of the products obtained from 1,3-dioxane structures at 170°C in 5 min after GVL-H_2O-H_2SO_4 treatment

4. Synthesis and NMR Spectra of products obtained from β-5

4.1 Phenylcoumarone 24

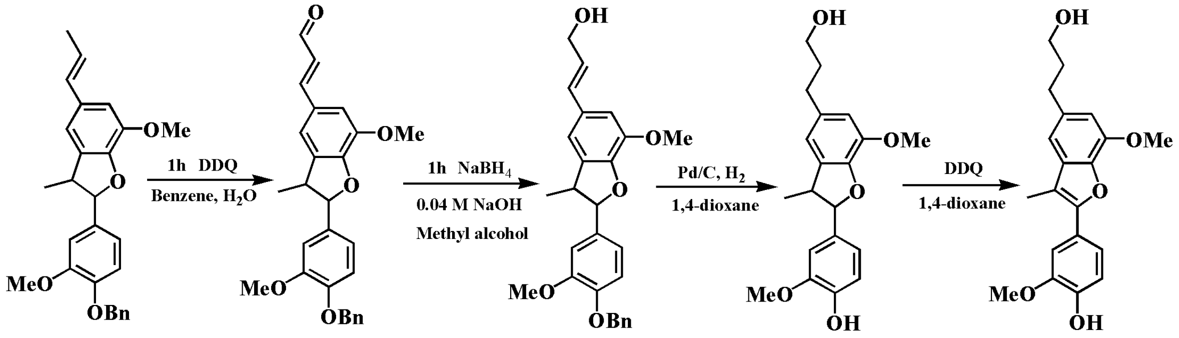

24

Fig.S15 Synthesis route of phenylcoumarone **24**

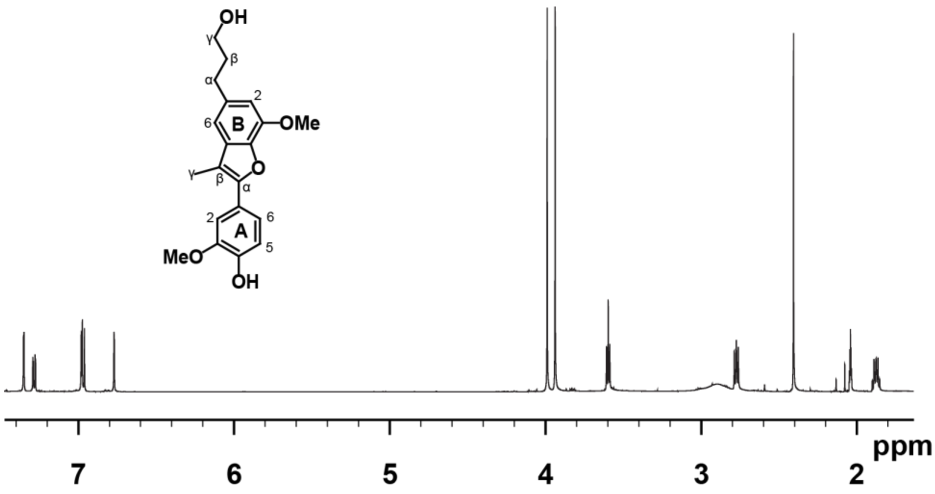

Fig.S16 ^{1}H NMR of phenylcoumarone **24**

24, ^{1}H-NMR (acetone-d6, 600MHz): δ 7.35 (d, J=2.0 Hz, 1H; A2), 7.28 (dd, J=2.0, 8.2 Hz, 1H; A6), 6.98 (t, J=1.2 Hz, 1H; B6), 6.97 (d, J= 8.2 Hz, 1H; A5), 6.77 (d, J=1.2Hz, 1H; B2), 3.99 (s, 3H; OCH_3), 3.94 (s, 3H; OCH_3), 3.60 (t, J=6.4Hz, 1H; Bγ-H), 2.77 (t, J=7.8Hz, 1H; Bα-H), 2.41 (s, 1H; Aγ-H), 1.91-1.85 (m, 1H; Bβ-H).

^{13}C NMR (acetone-d6, 600MHz): δ 151.98 (C-Aα), 148.52 (C-A3), 147.80 (C-A4), 145.64 (C-B3), 141.96 (C-B4), 138.65 (C-B1), 133.77 (C-B5), 124.04 (C-A1), 120.94 (C-A6), 116.16 (C-A5), 111.43 (C-B6), 110.87 (C-A2), 110.38 (C-Aβ), 108.55 (C-B2), 61.78(C-Bγ), 56.33 (C-OCH_3), 56.27 (C-OCH_3), 36.05 (C-Bβ), 33.21 (C-Bα), 9.64 (C-Aγ).

4.2 Stilbene 25

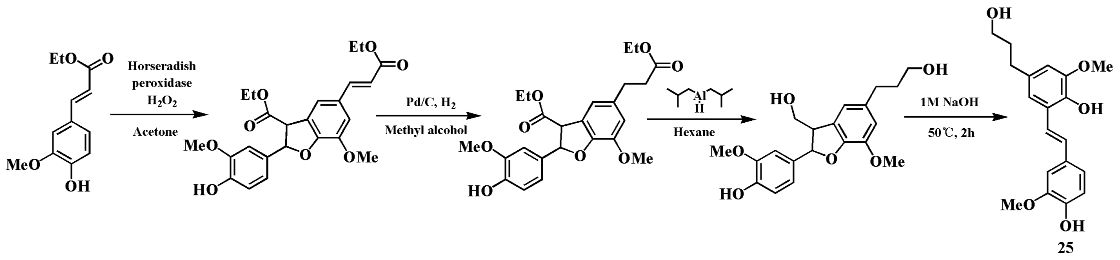

Fig.S17 Synthesis route of stilbene **25**

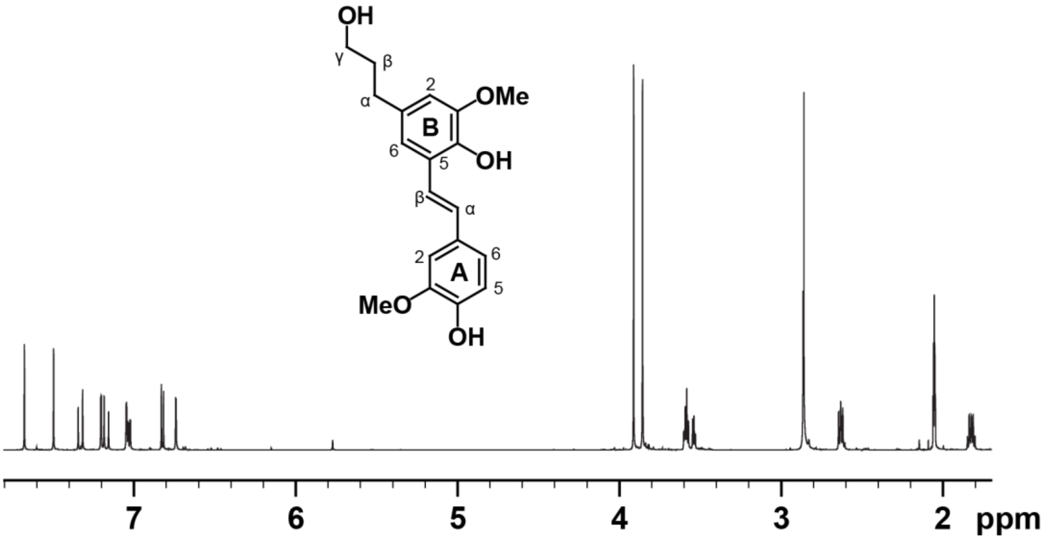

Fig.S18 ^{1}H NMR of stilbene **25**

25, ^{1}H-NMR (acetone-d6, 600MHz): δ 7.32 (d, J=16.5Hz, 1H; Aβ-H), 7.19 (d, J=1.9 Hz, 1H; A2), 7.16 (d, J=16.5Hz, 1H; Aα-H), 7.03 (d, J=1.7 Hz, 1H; B6), 7.01 (dd, J=1.9, 8.1 Hz, 1H; A6), 6.81 (d, J= 8.1 Hz, 1H; A5), 6.73 (1H, d, J=1.7Hz, H-B2), 3.90 (3H, s, H-OCH_3), 3.84 (3H, s, H-OCH_3), 3.60-3.55 (1H, m, H-Bγ), 2.62 (t, J=7.51Hz, 1H; Bα-H), 1.84-1.78 (m, 1H; Bγ-H).

^{13}C NMR (acetone-d6, 600MHz): δ 148.56 (C-A3), 148.24 (C-B3), 147.31 (C-A4), 142.85 (C-B4), 133.96 (C-B1), 131.19 (C-A1), 129.40 (C-Aα), 124.71 (C-B5), 121.72 (C-Aβ), 120.82 (C-A6), 118.58 (C-B6), 115.94 (C-A5), 111.04 (C-B2), 110.0 (C-A2), 61.78 (C-Bγ), 56.34 (C-OCH_3), 56.21 (C-OCH_3), 35.78 (C-Bβ), 32.65 (C-Bα).

4.3 Coumaran 27

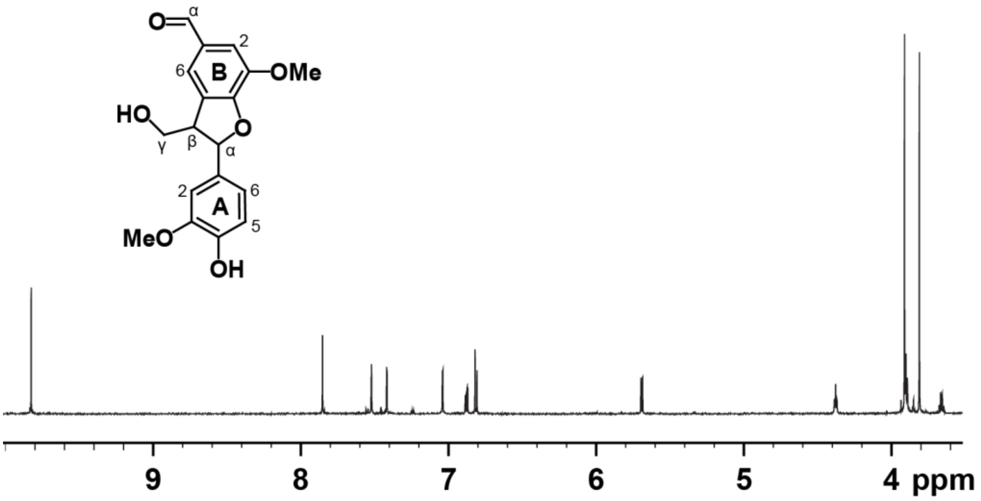

Fig.S19 ^{1}H NMR of benzoaldehyde coumaran **27**

27, ^{1}H-NMR (acetone-d6, 600MHz): δ 9.82 (s, 1H; Bα-H), 7.86 (s, 1H; ArOH-H), 7.52 (t, J=1.2 Hz, 1H; B6), 7.42 (d, J=1.4 Hz, 1H; B2), 7.04 (d, J=2.0 Hz, 1H; A2), 6.88 (dd, J=2.0, 8.1 Hz, 1H; A6), 6.81 (d, J=8.1Hz, 1H; A5), 5.69 (d, J=6.8Hz, 1H; Aα-H), 4.38 (t, 1H; γOH-H), 3.91 (s, 3H; OCH_3), 3.91-3.87 (m, 1H; Aγ-H), 3.81 (s, 3H; OCH_3), 3.68-3.63 (m, 1H; Aβ-H).

^{13}C NMR (acetone-d6, 600MHz): δ 191.05 (C-Bα), 154.83 (C-B4), 148.51 (C-A3), 147.67 (C-A4), 145.76 (C-B3), 133.31 (C-A1), 132.32 (C-B1), 131.14 (C-B5), 121.47 (C-B6), 119.74 (C-A6), 115.80 (C-A5), 113.34 (C-B2), 110.64 (C-A2), 89.85 (C-Aα), 64.15 (C-Aγ), 56.32 (C-OCH_3), 56.25 (C-OCH_3), 53.79 (C-Aβ).

4.4 Phenylcoumarone 29

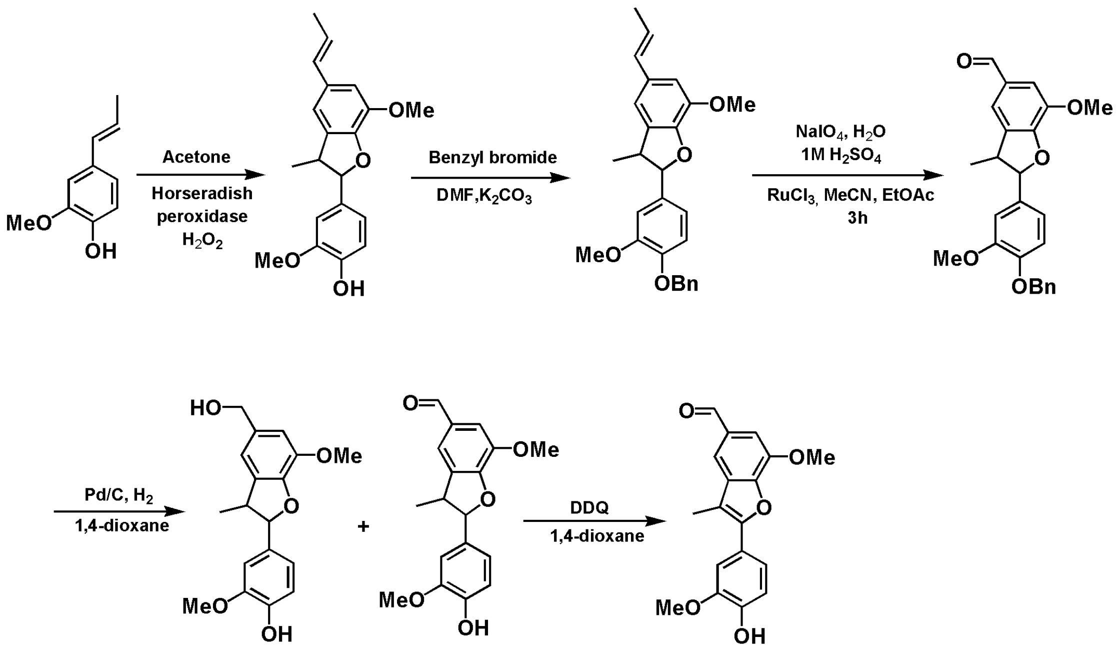

29

Fig.S20 Synthesis route of phenylcoumarone **29**

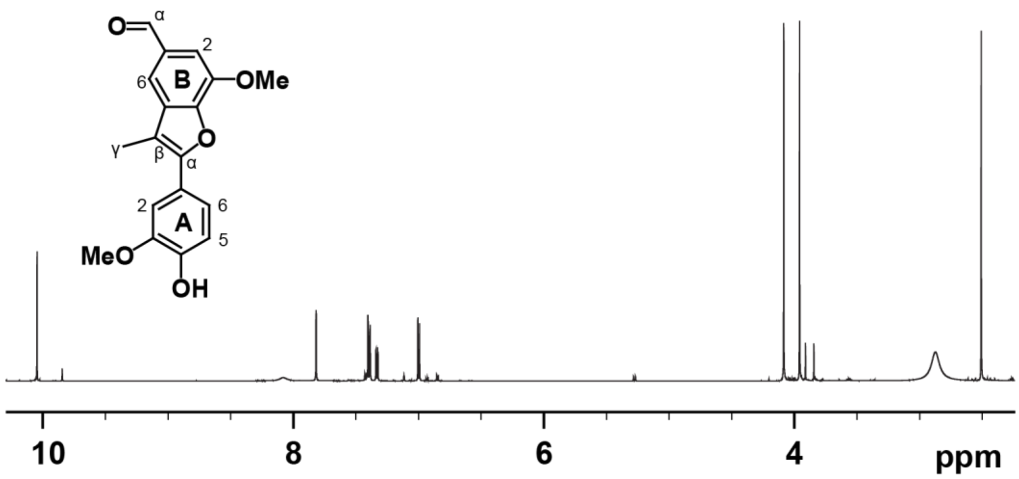

Fig.S21 ^{1}H NMR of phenylcoumarone **29**[18]

29, ^{1}H-NMR (acetone-d6, 600MHz): δ 10.05 (s, 1H; Bα-H), 7.82 (d, J=1.28 Hz, 1H; B6), 7.40 (d, J=1.2 Hz, 1H; B2), 7.38 (d, J=2.0 Hz, 1H; A2), 7.33 (dd, J=8.20, 2.0Hz, 1H; A6), 7.00 (d, J=8.2 Hz, 1H; A5), 4.08 (s,3H; OCH_3), 3.96 (s, 3H; OCH_3), 2.50 (s, 3H; CH_3).

4.5 Stilbene 30

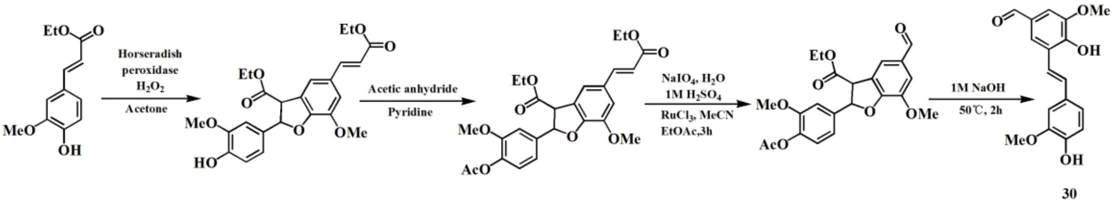

Fig.S22 Synthesis route of stilbene **30**

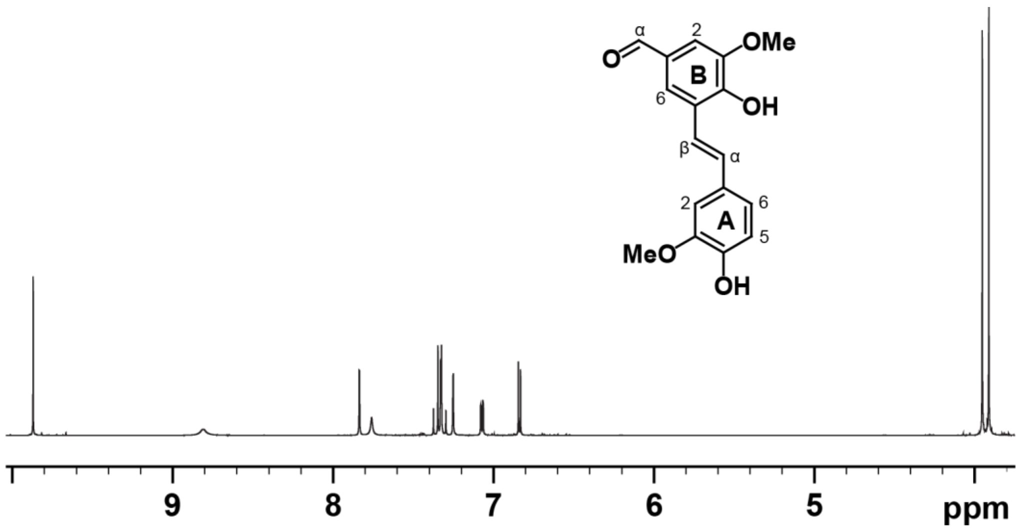

Fig.S23 ^{1}H NMR of stilbene **30**[18]

30, ^{1}H-NMR (acetone-d6, 600MHz): δ 9.87 (s, 1H; Bα-H), 7.83 (d, J=1.7 Hz, 1H; B6), 7.36 (d,

J=16.5 Hz, 1H; Aβ-H), 7.33 (d, J=1.9 Hz, 1H; B2), 7.31 (d, J=16.5 Hz, 1H; Aα-H), 7.25 (d, J=1.7 Hz, 1H; A2), 7.07 (dd, J=8.1, 1.9Hz, 1H; A6), 6.84 (d, J=8.13 Hz, 1H; A5), 3.95 (s, 3H; OCH_3), 3.91 (s, 3H; OCH_3).

^{13}C NMR (acetone-d6, 600MHz): δ 191.52 (C-Bα), 150.30 (C-B4), 149.07 (C-B3), 148.66 (C-A3), 147.80 (C-A4), 131.19 (C-Aα), 130.53 (C-A1), 130.00 (C-B1), 125.58 (C-B5), 124.06 (C-B6), 121.21 (C-A6), 120.07 (C-Aβ), 116.03 (C-A5), 110.22 (C-A2), 108.38 (C-B2), 56.50 (C-OCH_3), 56.22 (C-OCH_3).

5. Products obtained from syringaresinol (β-β)

5.1 Syringaresinol

31, ^{1}H-NMR (acetone-d6, 600MHz): δ 6.67 (s, 4H; A\B2\6), 4.66 (d, J=4.2 Hz, 2H; A\Bα-H), 4.21 (dd, J=6.9, 8.9 Hz, 2H; A\Bγ-H), 3.87-3.76 (m, 2H; A\Bγ-H), 3.87-3.76 (s,12H; OCH_3), 3.12-3.04 (m, 2H; Aβ-H).

^{13}C NMR (Acetone-d6, 600MHz): δ148.61 (C-A\B-3\5), 136.07 (C- A\B-4), 133.13 (C-A\B-1), 104.30 (C-A\B-2\6), 86.74 (C- A\B-α), 72.29 (C-A\B-γ), 56.55 (C-OCH_3), 56.53 (C-OCH_3), 55.26 (C- A\B-β).

5.2 Epi-syringaresinol

32, ^{1}H-NMR (acetone-d6, 600MHz): δ 6.68 (s, 4H; A\B2\6), 4.81 (d, J=6.0 Hz, 1H; Bα-H), 4.34 (d, J=7.0 Hz, 1H; Aα-H), 4.12 (m, 1H; Bγ-H), 3.87-3.76 (m, 2H; A\Bγ-H), 3.87-3.76 (s, 12H; OCH_3), 3.23-3.18 (m, 1H; Aγ-H), 3.51-3.47 (m, 1H; Aβ-H), 2.93-2.82 (m, 1H; Bβ-H).

^{13}C NMR (Acetone-d6, 600MHz): δ 148.59 (C-A\B-3\5), 148.49 (C-A\B-3\5), 136.13 (C-A\B-4), 135.56 (C-A\B-4), 133.24 (C-A\B-1), 130.32 (C-A\B-1), 104.29 (C-A\B-2\6), 103.89 (C-A\B-2\6), 88.65 (C-A\B-α), 82.74 (C-A\B-α), 71.43 (C-A\B-γ), 70.10 (C-A\B-γ), 56.55 (C-OCH_3), 56.53 (C-OCH_3), 55.65 (C- A\B-β), 50.19 (C- A\B-β).

5.3 Dia-syringaresinol

33, ^{1}H-NMR (acetone-d6, 600MHz): δ 6.69 (s, 4H; A\B2\6), 4.86 (d, J=4.9 Hz, 2H; A\Bα-H), 3.87-3.76 (s, 12H; OCH_3), 3.61-3.53 (m, 2H; A\Bγ-H), 3.51-3.47 (m, 2H; A\Bγ-H), 3.43-3.35 (m, 2H; Aβ-H).

^{13}C NMR (Acetone-d6, 600MHz): δ 147.03 (C-A\B-3\5), 135.70 (C-A\B-4), 130.85 (C-A\B-1), 104.54 (C-A\B-2\6), 84.77 (C-A\B-α), 69.23 (C-A\B-γ), 56.55 (C-OCH_3), 56.53 (C-OCH_3), 50.19 (C- A\B-β).

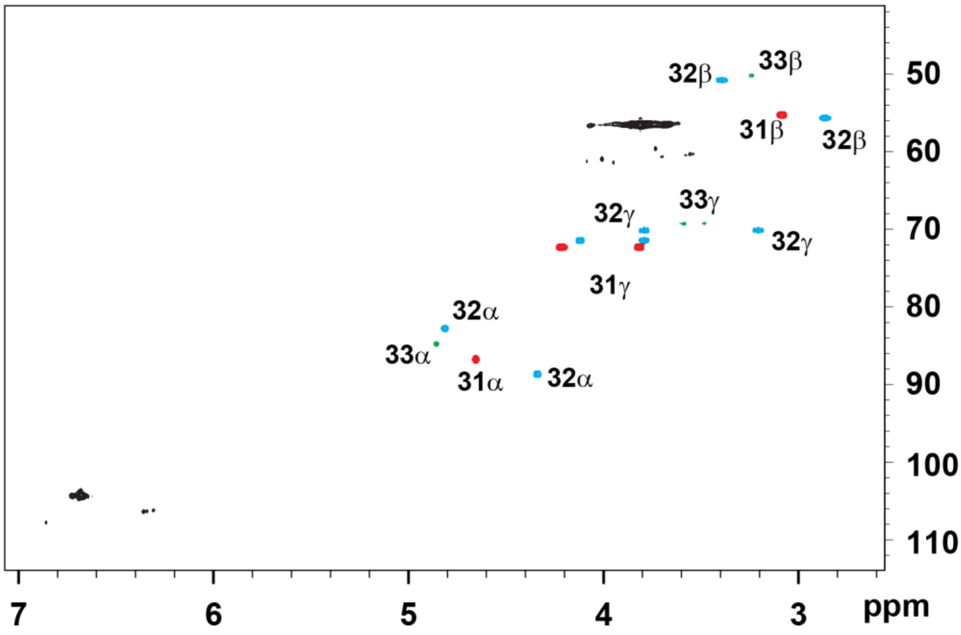

Fig.24 2D HSQC NMR spectrum of products of β-β after GVL-H_2O-H_2SO_4 treatment[19, 20]

5.4 GC-MS of the products obtained from β-β

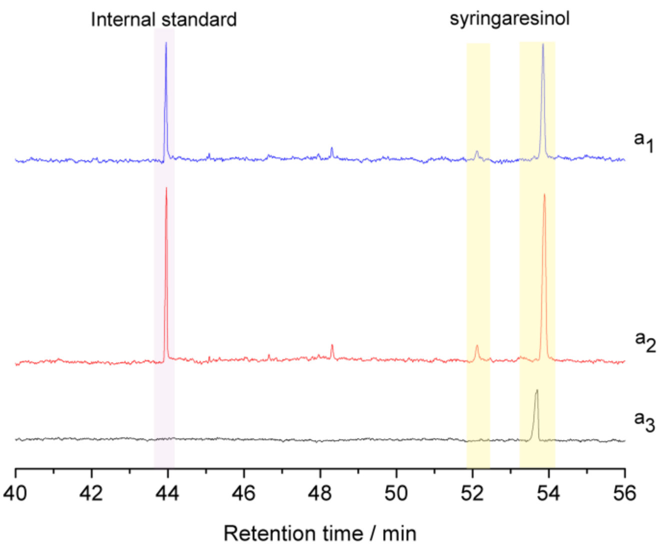

Fig.S25 GC-MS of the products obtained from β-β at 170°C after GVL-H_2O-H_2SO_4 treatment

a_1: products obtained from β-β in 60min; a_2: products obtained from β-β in 40min; a_3: β-β lignin model compound.

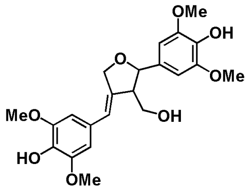

Fig.S26 The possible structure of the product eluted at retention time of 48.43 min

Based on the TMS-derived molecular ion of 632 for the peak at 48.43 (Fig.S24), we speculated the structure of the product is as showed in Fig.S26.

References

(1) Lundquist, K.; Hedlund, K. Acid degradation of lignin I: The formation of ketones of the guaiacylpropane series. *Acta Chem. Scand.* **1967,** *21* (7), 1750-1754.

(2) Lundquist, K.; Ericsson, L. Acid degradation of lignin III: Formation of formaldehyde. *Acta Chem. Scand.* **1970,** *24* (10), 3681-3686.

(3) Lundquist, K.; Kirk, T. K. Acid degradation of lignin IV: Analysis of lignin acidolysis products by gas chromatography, using trimethylsilyl derivatives. *Acta Chem. Scand.* **1971,** *25* (3), 889-894.

(4) Lundquist, K.; Ericsson, L. Acid degradation of lignin VI: Formation of methanol. *Acta Chem. Scand.* **1971,** *25* (2), 756-758.

(5) Lundquist, K.; Lundgren, R. Acid degradation of lignin part VII: The cleavage of ether bonds. *Acta Chem. Scand.* **1972,** *26* (5), 2005-2023.

(6) Yasuda, S.; Terashima, N.; Ito, T. Chemical structures of sulfuric acid lignin II: Chemical structures of condensation products from arylglycerol-β-aryl ether type structures. *Mokuzai Gakkaishi* **1981,** *27* (3), 216-222.

(7) Ito, T.; Terashima, N.; Yasuda, S. Chemical structures of sulfuric acid lignin III: Reaction of

arylglycerol-β-aryl ether with five percent sulfuric acid. *Mokuzai Gakkaishi* **1981,** *27* (6), 484-490.
(8) Hoo, L. H.; Sarkanen, K. V.; Anderson, C. D. Formation of C6C2-Enol Ethers in the Acid-Catalyzed Hydrolysis of Erythro-Veratrylglycerol-β-(2-Methoxyphenyl) Ether. *J. Wood Chem. Technol.* **1983,** *3* (2), 223-243.
(9) Yokoyama, T.; Matsumoto, Y. Revisiting the Mechanism ofβ-O-4 Bond Cleavage during Acidolysis of Lignin. Part 2: Detailed Reaction Mechanism of a Non-Phenolic C6-C2Type Model Compound. *J. Wood Chem.Technol.* **2010,** *30* (3), 269-282.
(10) Karlsson, O.; Lundquist, K.; Meuller, S.; Westlid, K. On the acidolytic cleavage of arylglycerol β-aryl ethers. *Acta Chem. Scand.* **1988,** *B42* (1), 48-51.
(11) Lundquist, K. Acid degradation of lignin II: Separation and identification of low molecular weight phenols. *Acta Chem. Scand.* **1970,** *24* (3), 889-907.
(12) Lee, D. Y.; Matsuoka, M.; Sumimoto, M. Mechanochemistry of lignin. IV. Mechanochemical reactions of phenylcoumaran models. *Holzforschung* **1990,** *44* (6), 415-418.
(13) Li, S.; Lundquist, K. Acid reactions of lignin models of β-5 type. *Holzforschung* **1999,** *53* (1), 39-42.
(14) Adler, E.; Lundquist, K. Spectrochemical estimation of phenylcoumaran elements in lignin. *Acta Chem. Scand.* **1963,** *17* (1), 13-26.
(15) Lundquist, K.; Hedlund, K. Acid degradation of lignin V: Degradation products related to the phenylcoumaran type of structure. *Acta Chem. Scand.* **1971,** *25* (6), 2199-2210.
(16) Ralph, J.; Young, R. A. Stereochemical Aspects Op Addition Reactions Involving Lignin Model Quinone Methides. *J. Wood Chem.Technol.* **2007,** *3* (2), 161-181.
(17) Lin, L.; Yao, Y.; Shiraishi, N. Liquefaction Mechanism of β-O-4 Lignin Model Compound in the Presence of Phenol under Acid Catalysis. Part 1. Identification of the Reaction Products. *Holzforschung* **2001,** *55* (6), 617-624.
(18) Lee, D. Y.; Matsuoka, M.; Sumimoto, M. Mechanochemistry of Lignin. IV. Mechanochemical Reactionsof Phenylcoumaran Models. *Holzforschung* **1990,** *44* (6), 415-418.
(19) Chen, C. Y.; Wu, T. Y.; Chang, F. R.; Wu, Y. C. Lignans and Kauranes from the Stems of Annona cherimola. *J. Chin. Chem. Soc.* **1998,** *45* (5), 629-634.
(20) Chang, F. R.; Chao, Y. C.; Teng, C. M.; Wu, Y. C. Chemical Constituents from Cassytha filiformis II. *J. Nat. Prod.* **1998,** *61* (7), 863-866.
